# Role of the Gut Microbiota and Its Metabolites in Tumorigenesis or Development of Colorectal Cancer

**DOI:** 10.1002/advs.202205563

**Published:** 2023-06-01

**Authors:** Ruize Qu, Yi Zhang, Yanpeng Ma, Xin Zhou, Lulu Sun, Changtao Jiang, Zhipeng Zhang, Wei Fu

**Affiliations:** ^1^ Department of General Surgery Peking University Third Hospital Beijing 100191 P. R. China; ^2^ Cancer Center Peking University Third Hospital Beijing 100191 P. R. China; ^3^ State Key Laboratory of Women's Reproductive Health and Fertility Promotion Peking University Beijing 100191 P. R. China; ^4^ Department of Endocrinology and Metabolism Peking University Third Hospital Beijing 100191 P. R. China; ^5^ Center of Basic Medical Research Institute of Medical Innovation and Research Third Hospital Peking University Beijing 100191 P. R. China; ^6^ Department of Physiology and Pathophysiology School of Basic Medical Sciences Peking University and the Key Laboratory of Molecular Cardiovascular Science (Peking University) Ministry of Education Beijing 100191 P. R. China; ^7^ Center for Obesity and Metabolic Disease Research School of Basic Medical Sciences Peking University Beijing 100191 P. R. China

**Keywords:** colorectal cancer, gut microbiota, intratumoral microbiota, metabolite, probiotic bacteria, tumorigenesis

## Abstract

Colorectal cancer (CRC) is the most common cancer of the digestive system with high mortality and morbidity rates. Gut microbiota is found in the intestines, especially the colorectum, and has structured crosstalk interactions with the host that affect several physiological processes. The gut microbiota include CRC‐promoting bacterial species, such as *Fusobacterium nucleatum*, *Escherichia coli*, and *Bacteroides fragilis*, and CRC‐protecting bacterial species, such as *Clostridium butyricum*, *Streptococcus thermophilus*, and *Lacticaseibacillus paracasei*, which along with other microorganisms, such as viruses and fungi, play critical roles in the development of CRC. Different bacterial features are identified in patients with early‐onset CRC, combined with different patterns between fecal and intratumoral microbiota. The gut microbiota may be beneficial in the diagnosis and treatment of CRC; some bacteria may serve as biomarkers while others as regulators of chemotherapy and immunotherapy. Furthermore, metabolites produced by the gut microbiota play essential roles in the crosstalk with CRC cells. Harmful metabolites include some primary bile acids and short‐chain fatty acids, whereas others, including ursodeoxycholic acid and butyrate, are beneficial and impede tumor development and progression. This review focuses on the gut microbiota and its metabolites, and their potential roles in the development, diagnosis, and treatment of CRC.

## Graphical abstract

The gut microbiota is associated with colorectal cancer (CRC), and identifying harmful or protective microorganisms and their metabolites in patients is of great significance in the diagnosis and treatment of CRC. Herein, the latest reports on the relationship between gut microbiota and metabolites in CRC and the prospects of treating CRC through targeting these metabolites are summarized and discussed.

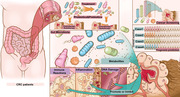

## 1 Introduction

Colorectal cancer (CRC) is the third most common and fatal cancer with 71 160 estimated new cases and 24 080 new deaths in the USA in 2023.^[^ ^1^ ^]^ CRC has heterogeneous genomic, epigenomic, and transcriptomic transformations and undergoes complex processes that lead to tumorigenesis.^[^ ^2^ ^]^ Genetic predisposition is an important factor in CRC progression accounting for 12–35% of all cases. According to this, CRC can be classified as Lynch syndrome, familial adenomatous polyposis, or Peutz‐Jeghers syndrome.^[^ ^3^ ^]^ Additionally, emerging evidence indicates that internal and external environmental factors are involved in the development of CRC.^[^ ^4^ ^]^ The heritability of CRC remains unclear in most patients, while sporadic CRC cases are mostly abided by the “adenoma–carcinoma sequence”; CRC evolves from colorectal polypoid adenomas and progresses into malignant tumors over several years, thus, suggesting the occurrence of complicated mutation procedures^[^ ^5^ ^]^ and illustrating the non‐negligible importance of environmental factors in its development and progression.^[^ ^6^ ^]^

The microenvironment of the human body is composed of a highly integrated microbiota, including bacteria, fungi, viruses, archaea, and parasites. Notably, the microbiota mainly consists of bacteria, particularly, *Firmicutes*, *Bacteroidetes*, and *Actinobacteria*.^[^ ^7^ ^]^ The colorectum has the largest population of bacteria, ≈3 × 10^13^ cells,^[^ ^8^ ^]^ and crosstalk between microorganisms and the colorectal epithelium is a key factor influencing basic physiological activities, including energy harvest, metabolic regulation, homeostatic preservation, and immune reactions.^[^ ^9^ ^]^ Dysbiosis‐related inflammation and carcinogen formation are two of the primary reasons for microbiota‐related carcinogenesis.^[^ ^10^ ^]^ Alterations of gut microbiota have been observed in precancerous adenomas during early cancer stages.^[^ ^11^ ^]^ Moreover, microorganisms, especially bacteria, are pivotal in the evolution of several diseases including CRC.^[^ ^12^ ^]^ Ratios of these microorganisms vary based on the anatomical site and between eastern and western countries,^[^ ^13^ ^]^ and these differences may be associated with disease development.^[^ ^14^ ^]^

Metabolites produced by gut microbiota, including bile acids (BAs) and short‐chain fatty acids (SCFAs), are essential components involved in the crosstalk between microorganisms and the host and play either protective or harmful roles in the development of CRC.^[^ ^15^ ^]^ The effects of these metabolites on inflammation and cancer development and progression warrant further investigation.^[^ ^16^ ^]^ Studies on the interaction between gut microbiota and CRC have been conducted and hence should be systematically summarized. Moreover, other gut microbiota components, such as viruses and fungi, the recently discovered probiotics from gut microbiota that related to CRC, the intratumoral microbiota in CRC, the pattern of gut microbiota in early‐onset CRC patients, and the metabolites produced by the microbiota have also been reviewed, but to a lesser extent. In this review, we summarize and discuss the latest reports on the relationship between gut microbiota and metabolites in CRC and the prospects of treating CRC through the targeting of these metabolites.

## 2 Association between Gut Microbiota and CRC

### 2.1 Landscape of CRC Microbiota

The gut microbiota of healthy individuals is less diverse than that of patients with CRC, which show a global compositional shift.^[^ ^17^ ^]^ Specific bacteria, including *Fusobacterium nucleatum*, *Escherichia coli*, *Bacteroides fragilis*, and *Streptococcus gallolyticus*, were linked to CRC in various association and mechanistic studies.^[^ ^18^ ^]^ Despite geographical variations in gut microbiota, a microbial core of seven enriched bacteria (*B. fragilis*, *F. nucleatum*, *Porphyromonas asaccharolytica*, *Parvimonas micra*, *Prevotella intermedia*, *Alistipes finegoldii*, and *Thermanaerovibrio acidaminovorans*) has been confirmed in CRC through meta‐analysis of 526 fecal samples from shotgun metagenome datasets.^[^ ^19^ ^]^ Therefore, microbiota can be used as CRC biomarkers through the detection and quantification of the alterations in the relative abundances of these bacteria.

Gut microbiota composition varies between anatomical locations.^[^ ^20^ ^]^ As was previously reported, operational taxonomic unit level of gut microbiota composition changed in distal but not proximal CRC cases, while mucosal bacterial biofilms affects tumor formation in proximal CRC cases but not distal ones, suggesting different roles of gut microbiota in different anatomical subsites of CRC.^[^ ^21^ ^]^ One reason could be that bacteria showed more accumulation in the distal colon when there are changes in the local environment including surgery and infection;^[^ ^22^ ^]^ it was also found that bacterial oncogenes were more abundant in the stools of distal CRC patients compared to proximal CRC patients.^[^ ^23^ ^]^ However, a greater abundance of *F. nucleatum* was found in proximal CRC cases, whose metabolism can induce carcinogens and inflammation in the tumor microenvironment and increase mutation rate.^[^ ^24^ ^]^ These phenomena suggest that the subsite location of CRC tumor may be associated with site‐specific bacteria, and some bacteria play differential roles in proximal and distal CRCs, which should be further examined in the future.

Other than bacteria, the gut microbiota also includes viruses and fungi whose roles in the development of CRC have been studied. One study indicated substantial changes in the abundance of enteroviruses and 22 other viral taxa (including cytomegalovirus) in patients with CRC,^[^ ^25^ ^]^ but other studies have reported conflicting data.^[^ ^26^ ^]^ Meanwhile, another study highlighted the changes in phage characteristics in CRC and indicated that the cancer‐related virome is primarily formed by temperate bacteriophages.^[^ ^27^ ^]^ Despite limited studies, an enhanced abundance of *Malassezia* has been identified in patients with CRC.^[^ ^28^ ^]^ These studies suggest that transkingdom microbial interactions may be essential in CRC oncogenesis.

### 2.2 Interaction between Gut Microbiota and CRC

Subsequent studies have shown that the gut microbiota which includes *E. coli*, *Enterococcus*, *Bacteroides*, and *Clostridium* can contribute to CRC by enhancing abnormal crypt foci induced by 1,2‐dimethylhydrazine.^[^ ^29^ ^]^ Interestingly, mice transplanted with gut microbiota from patients with CRC had more intestinal polyps than those receiving microbiota from healthy controls.^[^ ^30^ ^]^ Comparative metagenomic or metataxonomic approaches have indisputably proven that patients with CRC have an imbalanced taxonomic structure in comparison to healthy individuals, with an upregulation and downregulation in the abundance of pro‐oncogenic (e.g., *Bacteroides*, *Escherichia*, *Fusobacterium*, and *Porphyromonas*) and potentially protective (e.g., *Roseburia*) bacteria, respectively.^[^ ^31^ ^]^ These data highlight the essential role of the microbial communities on carcinogenesis.

Undeniably, the formation of the human gut microbiota is continuously progressing, but the functional components of this microbiota are not well understood. This could be attributed to the complex composition of the gut microbiota and the impact of the external environment on the microbes.

**Figure 1:**
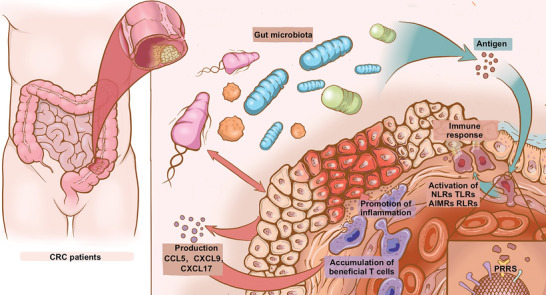
The role of the gut microbiota in CRC progression through immune, inflammatory, and metabolic reactions. Several bacterial species in the gut could induce the host to produce chemokines including chemokine (C‐C motif) ligand 5 (CCL5), chemokine (C‐X‐C motif) ligands 9 (CXCL9), and CXCL17. Tumor cells accumulate T cells that promote inflammation and leads to progression of CRC. Microbial antigens produced by the gut microbiota could activate signaling through various receptors including nucleotide‐binding oligomerization‐like receptor (NLR), retinoic acid‐inducible gene I‐like receptor (RLR), toll‐like receptor (TLR), and absent in melanoma 2‐like receptor (AIMR), that stimulate the immune response.

These factors result in unpredictable crosstalk between the host and microbes.^[^ ^32^ ^]^ Host–microbial interactions in CRC were first identified in 1975, when it was shown that the incidence of colon tumors caused by dimethylhydrazine, a carcinogen, was significantly lower in germ‐free rats than in rats with an intestinal microbiome.^[^ ^4^ ^]^ Notably, the influence of the gut microbiota on the development of CRC includes communication with the host immune system through pattern recognition receptor signaling, inflammasomes, and cytokines; the production of inflammation, which is a special type of immune reaction; and the secretion of metabolites, including BAs and SCFAs.^[^ ^33^ ^]^ Mechanistically, inflammatory and immune reactions caused by the interplay between the CRC‐promoting bacteria and the host are among the most common mechanisms in CRC development^[^ ^34^ ^]^ (**Figure** **1**). These bacterial species stimulate chemokine production by CRC cells including chemokine (C‐C motif) ligand 5 and chemokine (C‐X‐C motif) ligands 9 and 17, thereby activating immune responses which can lead to accumulation of T cells (e.g., cytotoxic T lymphocytes, T helper type 1 (Th1), and interleukin (IL)‐17‐producing Th cells) in tumor tissues^[^ ^35^ ^]^ and the modulation of inflammatory responses in the colon. The latter occurs through cytokine secretion, specifically of IL‐17A, IL‐22, and IL‐23A, which promote tissue inflammation.^[^ ^30^ ^]^ Moreover, pattern recognition receptors are involved in the crosstalk between the immune system and the microbiota as they can recognize microbial antigens and activate a number of signaling pathways, including the nucleotide‐binding oligomerization domain‐like receptor,^[^ ^36^ ^]^ retinoic acid‐inducible gene I‐like receptor,^[^ ^37^ ^]^ toll‐like receptor,^[^ ^38^ ^]^ and absent in melanoma 2‐like receptor^[^ ^39^ ^]^ pathways, to activate an immune response. Furthermore, production of metabolites that induce CRC development is another essential mechanism. However, the detailed mechanism of host–microbial interaction in CRC is still unknown, and further confirmatory research is needed before it can be leveraged for beneficial therapeutic effects.

Herein, we discuss the major bacterial species of the microbiota and summarize the protective or harmful roles they may play in CRC formation, development, and/or metastasis (**Table** **1**).

**Table 1:** Mechanisms of action of CRC‐associated gut bacterial species

Organism	Gram staining	Effect in CRC	Expression pattern in CRC	Mechanism	Role in tumor formation, development, and metastasis	Interaction with other gut microbiota
*Fusobacterium nucleatum*	Negative	Promote	Enhanced	Proliferation of CRC cells DNA damage and carcinogenesis through oxidative stress Mutation in the Apc tumor suppressor gene Increase of tumor‐promoting cytokines through NF‐*κ*B activation Secretion of FadA adhesion protein that activating Wnt/*β*‐catenin signaling Inhibit T cell activation and NK cell‐meditated killing of tumor cells Immune surveillance suppression Promote CRC cell to acquire stem cell‐like features	Formation Development Metastasis	–
*Escherichia coli*	Negative	Promote	Enhanced	Inflammatory reactions DNA damage Secretion of virulence factors to activate COX‐2, etc. Promote cell‐cycle arrest Induce apoptosis of CRC cells Disrupt the gut vascular barrier	Formation Metastasis	Promoting procarcinogenic bacteria including *Bacteroides fragilis*
*Bacteroides fragilis*	Negative	Promote	Enhanced Higher in proximal colon	Interaction with IL‐17 Activation of NF‐*κ*B Accumulation of regulatory T cells to enhance inflammatory response Increasing paracellular permeability Activation of *β*‐catenin signaling pathway Proliferation of CRC cells DNA damage through polyamine metabolism Inducing bacterial dysbiosis by promoting procarcinogenic bacteria Disturbance of the host immune apparatus and gut barrier Promotion of mucin degradation	Development	Promoting procarcinogenic bacteria including *Escherichia coli*
*Streptococcus gallolyticus*	Positive	Promote	Enhanced	Activation of NF‐*κ*B and IL‐8 Inflammatory reaction Proliferation of CRC cells through *β*‐catenin signaling but not inflammation	Formation	–
*Clostridium butyricum*	Positive	Inhibit	Decreased	Production of SCFAs including butyrate and acetate Decrease of secondary BAs secretion Inhibiting proliferation of CRC cells Inducing apoptosis in CRC cells Inhibition of Wnt/*β*‐catenin signaling pathway Modulation of gut microbiota Activation of GPRs including GPR43 and GPR109A	Development	Modulating gut microbiota
*Streptococcus thermophilus*	Positive	Inhibit	Decreased	Banning the invasion of *Escherichia coli* Production of *β*‐Galactosidase to enhance abundance of probiotics Activation of oxidative phosphorylation Inhibition of Hippo signaling pathway	Development	Banning the invasion of *Escherichia coli* Enhance abundance of probiotics including *Bifidobacterium* and *Lactobacillus*
*Lacticaseibacillus paracasei*	Positive	Inhibit	Decreased	Inhibiting proliferation of CRC cells Inducing apoptosis of CRC cells Producing LpEVs as intercellular communication molecules *Lactobacillus paracasei* DTA81 diminish liver oxidative stress and decrease proliferating cell nuclear antigen	Development	–
*Ruminococcus gnavus*	Positive	Inhibit	Decreased	Degrade lysoglycerophospholipid Promote tumor immune surveillance function of CD8^+^T cell	Formation Development	Commensal growing with *Blautia producta*

CRC: colorectal cancer; NF‐*κ*B: nuclear factor‐kappa B; NK: natural killer; COX‐2: cyclooxygenase 2; IL‐: interleukin; GPR: G‐protein coupled receptor; LpEVs: Lacticaseibacillus paracasei‐derived extracellular vesicles.

#### 2.2.1 CRC‐Promoting Bacterial Species

Enrichment of tumor‐promoting bacterial species is an important mechanism employed by gut microbiota during CRC development. Along with polyketide synthase positive (pks^+^) *E. coli*, enterotoxigenic *B. fragilis*, and *S. gallolyticus*, *F. nucleatum* isolates are major protumorigenic bacteria. *F. nucleatum* is a gram‐negative oral commensal anaerobe implicated in a wide spectrum of disorders, such as cardiovascular diseases, rheumatoid arthritis, inflammatory bowel disease, and CRC.^[^ ^40^ ^]^ Patients with these disorders show higher levels of *F. nucleatum* compared to healthy individuals;^[^ ^41^ ^]^ importantly, a progressive trend for an increased abundance of *F. nucleatum* in CRC, from highly dysplastic adenomas through to late cancer stages, has been observed.^[^ ^5^ ^]^ *F. nucleatum* can activate the formation and development of CRC through proliferation of cancer cells but not noncancerous cell lines.^[^ ^42^ ^]^ Infection of CRC cells with *F. nucleatum* increases tumor cell proliferation, invasive activity, and ability to form xenograft tumors in Apc^min/+^ mice.^[^ ^43^ ^]^ The abundance of *F. nucleatum* in CRC was associated with an increase of tumor‐promoting cytokines, including IL‐17A and tumor necrosis factor‐*α* (TNF‐*α*), through the sensitization of the nuclear factor‐kappa B (NF‐*κ*B) signaling pathway.^[^ ^44^ ^]^ Additionally, *F. nucleatum has been shown* to promote CRC metastasis by upregulating long noncoding RNA Keratin7‐antisense (KRT7‐AS) and Keratin7 (KRT7) through activation of NF‐*κ*B.^[^ ^45^ ^]^ *F. nucleatum* also causes CRC cells to acquire stem cell‐like features through manipulating lipid droplet‐mediated Numb degradation.^[^ ^46^ ^]^ The FadA adhesion protein secreted by *F. nucleatum* binds to E‐cadherin, and enhances the level of inflammatory and cancer genes, through activating the Wnt/*β*‐catenin signaling in intestinal epithelial cells.^[^ ^42^, ^47^ ^]^

*E. coli* is a gram‐negative anaerobic bacterium mainly found in the intestines. Though generally not pathogenic, some virulent strains of *E. coli* which colonize the human gut are nosogenic and can lead to diseases via multiple mechanisms.^[^ ^48^ ^]^ Greater abundance of *E. coli* was observed in mucosal samples from patients with CRC.^[^ ^49^ ^]^ Colibactin, a genotoxic polyketide‐peptide produced by polyketide synthase positive (pks^+^) *E. coli*,^[^ ^50^ ^]^ causes double‐stranded DNA damage through the activation of the DNA damage signaling and cell‐cycle arrest pathways^[^ ^51^ ^]^ and induction of tumor formation.^[^ ^52^ ^]^ *E. coli* also secretes other virulence factors, such as cycle‐inhibiting factor, cytotoxic necrotizing factor and cyclooxygenase 2 (COX‐2), and cytolethal distending toxin, which can induce cell‐cycle arrest, DNA damage, and apoptosis.^[^ ^53^ ^]^ Moreover, *E. coli* was also found to promote CRC metastasis through disrupting the gut vascular barrier, and induce the formation of a premetastatic niche.^[^ ^54^ ^]^

*B. fragilis* toxin (BFT), a secreted metalloproteinase enterotoxin, is linked to early carcinogenesis. BFT is associated with the occurrence of adenomas and serrated polyps in the human colon,^[^ ^11a^ ^]^ promotion of Th17‐mediated colitis in a mouse model,^[^ ^55^ ^]^ and distal CRC in Apc*^min+/−^* mice through interaction with IL‐17 and subsequent activation of the NF‐*κ*B signaling pathway.^[^ ^56^ ^]^ In addition, colonization by BFT^+^ *B. fragilis* can promote accumulation of regulatory T cells, thereby inducing IL‐17‐mediated procarcinogenic inflammatory responses.^[^ ^57^ ^]^ In colon epithelial cells, BFT promotes the cleavage of E‐cadherin, increasing paracellular permeability and activating the *β*‐catenin signaling pathway leading to enhanced cell proliferation.^[^ ^58^ ^]^ Moreover, BFT activates DNA damage through polyamine catabolism in CRC cells.^[^ ^59^ ^]^ Interestingly, BFT^+^ *B. fragilis* can also induce bacterial dysbiosis locally by promoting the growth of other procarcinogenic bacteria, disturbing the host immune apparatus^[^ ^57^ ^]^ and the gut barrier,^[^ ^58^ ^]^ and inducing mucin degradation.^[^ ^60^ ^]^

*S. gallolyticus* infection‐related endocarditis and bacteremia can enhance the risk of CRC,^[^ ^61^ ^]^ and exposure to *S. gallolyticus* antigens is closely correlated with increased CRC incidence.^[^ ^62^ ^]^ In xenograft and azoxymethane/dextran sodium sulfate (AOM/DSS) mouse models exposed to *S. gallolyticus*, the tumor burden increased, and the dysplasia grade worsened.^[^ ^63^ ^]^ Nevertheless, the underlying mechanisms involved in *S. gallolyticus‐*mediated CRC progression may be associated with high levels of NF‐*κ*B and IL‐8 mRNA, inflammatory reactions, and *β*‐catenin,^[^ ^63a^ ^]^ although further investigations are warranted.

A meta‐analysis has revealed seven bacteria commonly enriched in CRC,^[^ ^19^ ^]^ including *P. asaccharolytica*, *P. micra*, *P. intermedia*, *A. finegoldii*, and *T. acidaminovorans*. These bacterial species may have harmful roles in cancer promotion, yet their roles in CRC progression have not been adequately studied to date.

#### 2.2.2 CRC‐Protecting Bacterial Species

In addition to these core bacteria, several CRC‐depleted bacteria have been named and were negatively linked to mutualistic networks of enriched bacteria.^[^ ^19^ ^]^ The depleted bacteria included probiotic bacteria, such as *Clostridium butyricum* and *Streptococcus thermophilus*, which are potentially beneficial in CRC prevention through production of substances, such as butyrate, and antagonistic action against the pathobionts.^[^ ^64^ ^]^ *Ruminococcus gnavus* and *Blautia producta* are commensal bacteria that were found to degrade lysoglycerophospholipid in the microenvironment of the intestinal tissue and promote tumor immune surveillance function of CD8^+^T cells, thus inhibiting the occurrence and development of colon cancer.^[^ ^65^ ^]^ Research on CRC‐protecting microbiota is still limited, despite its high translational potential in prevention and treatment of CRC.

##### Clostridium Butyricum

*C. butyricum*, a gram‐positive anaerobic bacterium that produces butyrate, exists in various natural environments, including soil, milk products, and some vegetables. *C. butyricum* is also one of the common human gut microbiota species that is found in ≈10% to 20% of adults and is one of the original colonizers found in human infants.^[^ ^66^ ^]^ As a symbiont in the human digestive system, *C. butyricum* ferments fibrous foods in the gut to aid digestion, thus, producing SCFAs, including butyrate and acetate,^[^ ^67^ ^]^ which are protective in CRC. In addition, *C. butyricum* inhibits proliferation and induces apoptosis in CRC cells, through interacting with the Wnt/*β*‐catenin signaling pathway and modulating the composition of the gut microbiota. This decreases the secretion of secondary BAs closely related to cancer development, enhances the secretion of SCFAs, and activates G‐protein coupled receptors (GPRs) including GPR43 and GPR109A that suppress tumor progression.^[^ ^68^ ^]^

##### Streptococcus Thermophilus

*S. thermophilus* is a gram‐positive fermentative anaerobic bacterium which can be isolated from fermented milk products, such as yogurt. It protects the gut epithelium through preventing invasive *E. coli*, thereby relieving acute diarrhea in infants, and small‐intestinal methotrexate‐induced mucositis in rat models.^[^ ^69^ ^]^ Gavage of *S. thermophilus* inhibited tumor formation in both Apc^min/+^ and azoxymethane‐injected mice. Moreover, production of *β*‐galactosidase by *S. thermophilus* enhances the abundance of probiotics, such as *Bifidobacterium* and *Lactobacillus*. Furthermore, the galactose produced by *S. thermophilus* was shown to modulate oxidative phosphorylation and the Hippo signaling pathway, which lead to tumor‐suppressive effects.^[^ ^70^ ^]^

##### Lacticaseibacillus Paracasei

*L. paracasei* is a gram‐positive bacterium with probiotic properties in CRC. It enhances apoptosis and inhibits proliferation of tumor cells by regulating the Bcl‐2 family proteins, producing reactive oxygen species (ROS) and cell wall protein, enhancing calreticulin translocation, and modifying the cell cycle.^[^ ^71^ ^]^ *L. paracasei*‐derived extracellular vesicles act as intercellular communication molecules. They are taken up by CRC cells, where they inhibit the proliferation, migration, invasion, and promote apoptosis.^[^ ^72^ ^]^ Additionally, *L. paracasei* DTA81 prevents the development of CRC at an early stage by diminishing liver oxidative stress (OS) and decreasing the secretion of proliferating cell nuclear antigen.^[^ ^73^ ^]^

#### 2.2.3 Other Microbiota Types

##### Virome

Alterations have been reported in the fecal virome (phages included) in patients with CRC.^[^ ^25^, ^27^ ^]^ The bacterial composition of the gut microbiota could be changed through phage regulation including pathogenic bacteria development and subsequent biofilm formation. An increase in the abundance of several phages was found in the feces of patients with CRC. Through analysis of 317 metagenomic samples gathered from China (Hong Kong), Austria, and Japan, enrichment of five bacteriophages was identified, namely, *Peptacetobacter hiranonis* Phage, *F. nucleatum animalis 7_1* Phage, *F. nucleatum polymorphum* Phage, *F. nucleatum animalis 4_8* Phage, and *P. micra* Phage.^[^ ^74^ ^]^ *SpSL1* is the phage that being more abundant in early‐stage CRC, and infects *Streptococcus* species.^[^ ^25^ ^]^ *HK544*, *Punalikevirus*, *Lambdalikevirus*, and *Mulikevirus* can reduce the abundance of *E. coli* in patients with CRC and lead to dysbiosis.^[^ ^25^ ^]^ However, the causal relationship between the phage and bacteria remains unclear, and further studies on the underlying mechanisms are essential to identify potential therapeutic targets.

##### Fungi

Fungi are major components of the gut microbiota whose dysbiosis affects human health through induction of inflammatory and immune reactions.^[^ ^75^ ^]^ Fungal diversity is diminished in patients with CRC in comparison to healthy individuals, which is more evident in the early stage the disease.^[^ ^76^ ^]^ Dysbiosis in patients with CRC and colon polyps could be attributed to the downregulation of beneficial yeasts and upregulation of pathological fungi, including *Trichosporon* and *Malassezia genera*. Immune reaction is currently considered to be the major paths employed by fungi during CRC development. For instance, *Candida albicans* can activate the interaction between macrophages and innate lymphoid cells, increase expression of IL‐22, and promote CRC.^[^ ^77^ ^]^ Dysbiosis of gut fungi also affects the function of CARD9, thereby decreasing the activation of inflammasomes and maturation of IL‐18 and enhancing expansion of myeloid‐derived suppressor cells. Consequently, this increases susceptibility to CRC.^[^ ^75^ ^]^ Owing to the limited studies and sample sizes, the spectrum of fungi in CRC remains unclear, although fungal dysbiosis has important roles in tumor development.

### 2.3 Intratumoral Microbiota in CRC

The advent of metagenomic sequencing highlights the tumor microbiota as an integral part of the microenvironment affecting tumor progression. Moreover, the abundance of *Fusobacterium* increased in liver metastatic CRCs but not primary liver cancers,^[^ ^78^ ^]^ and intratumoral *E. coli* damaged the gut vascular barrier and promoted metastasis,^[^ ^54^ ^]^ indicating the importance of tumor microbiota. The intratumoral microbiota is heterogeneous and progressively changes as CRC progresses from adenoma to carcinoma.^[^ ^79^ ^]^ The mechanisms underlying these changes are unclear. One study evaluating CRC and adenoma samples found an enrichment of *Fusobacterium*, *Bacteroides*, *Parvimonas*, and *Prevotella* without a reduction in other bacteria.^[^ ^79^ ^]^ Yet another study, which examined 294 matched tumor and tumor‐adjacent biopsies, reported an increase in the abundance of *Fusobacterium*, *Streptococcus*, and *Proteobacteria* in tumors, whereas *Firmicutes* abundance decreased.^[^ ^80^ ^]^ Interestingly, the intratumoral microbiota also changes based on the anatomical subsite; ascending and descending colon cancer samples showed significant microbial alterations.^[^ ^81^ ^]^ Importantly, the microbiota in a single tumor is evenly distributed and not limited to some regions.^[^ ^82^ ^]^

*F. nucleatum* is among the most common bacterial species in patients with CRC and can be detected in their biopsies. Its DNA is generally found in tumor cells.^[^ ^83^ ^]^ Moreover, increased levels of mucosa‐associated and internalized *E. coli* were observed in tumors, and higher bacterial levels were observed in patients with stage III/IV cancer than in those with stage I cancer, suggesting a possible association between bacterial abundance and poor prognosis.^[^ ^54^ ^]^ Data on intratumoral microbiota in CRC are currently limited, but a list of available literature sources has been summarized in **Table** **2**. Future research on the microbial community of tumors may further reveal the host–bacterial interactions in CRC.

**Table 2:** Summary of recent studies on intratumoral microbiota in CRC

					Gut microbiota	
Author	Year	Disease type	Sample type	Sample size	Increase	Decrease
Burns	2015	CRC	Surgery tissue	88	*Candidatus* *Portiera* *Providencia* *Fusobacterium*	*Bacteroides* *Rikenellaceae* *Lachnospiraceae* *Ruminococcaceae*
Flemer	2017	CRC Colorectal polyp	Surgery tissue Biopsies tissue	136	*Bacteroidetes* Cluster2 *Firmicutes* Cluster2 *Pathogen* *Prevotella*	*Bacteroidetes* Cluster1 *Firmicutes* Cluster1
Bullman	2017	CRC	Fresh‐frozen tissue	88	*Fusobacterium nucleatum* *Fusobacterium necrophorum* *Bacteroides fragilis* *Bacteroides thetaiotaomicron*	None
Shah	2018	CRC	Biopsies tissue	294	*Fusobacterium* *Streptococcus* *Parvimonas*	*Faecalibacterium* *Ruminococcaceae*
Nielsen	2019	CRC Colorectal adenoma	FFPE tissue	299	*Prevotella*	None
Lee	2021	CRC (MSI‐H)	Surgery tissue	153	*Fusobacterium nucleatum*	None
Kordahi	2021	Colorectal polyps	Biopsies tissue	240	TAP: *Bacteroidetes* SSP: *Proteobacteria*	*Firmicutes*
Liu	2021	CRC Colorectal adenoma	Biopsies tissue	436	*Fusobacterium* *Bacteroides* *Parvimonas* *Prevotella*	None

CRC: colorectal cancer; MSI‐H: microsatellite instability high; TAP: tubular adenomatous polyps; SSP: sessile serrated polyps; FFPE: formalin‐fixed and paraffin‐embedded.

## 3 Gut Microbiota Metabolites and Their Effects on CRC

**Figure 2:**
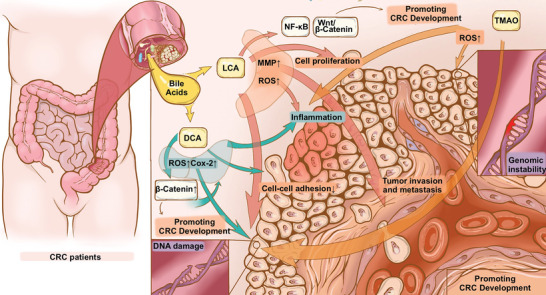
Promoting effects of gut microbiota metabolites on CRC. Metabolites including several bile acids (e.g., DCA and LCA), and trimethylamine n‐oxide (TMAO) promote CRC development by increasing cancer cell proliferation, increasing DNA damage, enhancing tumor invasion and metastasis, reducing cell–cell adhesion, and promoting genomic instability.

**Figure 3:**
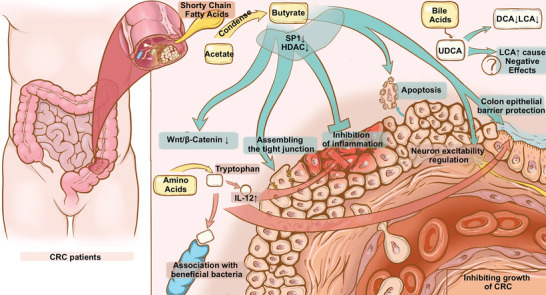
Protective roles of gut microbiota metabolites against CRC. Metabolites including bile acids such as ursodeoxycholic acid (UDCA), SCFAs such as butyrate, and amino acids play a protective role by assembling the tight junction, inhibiting inflammation, promoting cancer cell apoptosis, and protecting colon epithelial barriers, to inhibit growth of CRC.

Metabolites are essential components in the interaction (crosstalk) between the gut microbiota and the human body.^[^ ^6^ ^]^ Microorganisms in the colon produce metabolites after the uptake of certain substances,^[^ ^84^ ^]^ which then act as signaling elements and substrates in metabolic reactions^[^ ^85^ ^]^ and are closely related to cancer development, including CRC.^[^ ^86^ ^]^ The composition of gut microbiota metabolites includes volatile small molecules, lipids, proteins and peptides, sugars, secondary bile products or terpenes, biogenic amines, oligosaccharides, glycolipids, organic acids, and amino acids.^[^ ^53^ ^]^ Various metabolites possess different properties depending on the regulation pathways involved; some have health‐promoting and antineoplastic abilities, while others have proinflammatory and carcinogenic properties.^[^ ^87^ ^]^ Additionally, these metabolites are potentially beneficial in clinical and translational research, particularly, in the detection of cancers such as CRC and prediction of treatment effects.^[^ ^88^ ^]^ Moreover, they may be more easily applicable than microbial taxa because they affect microbial functions and host–microbiota metabolic crosstalk.^[^ ^85^ ^]^ In the following sections, we summarized some major gut microbiota metabolites and their promoting (**Figure** **2**) and inhibiting (**Figure** **3**) effects on CRC (**Table** **3**).

**Table 3:** Gut microbiota‐associated metabolites in CRC and their mechanisms of action

Class of metabolites	Metabolite	Producers	Potential mechanism
BAs	DCA	Clostridium hiranonis, Clostridium hylemonae, Clostridium sordelli, Clostridium scindens	Inflammation, angiogenic formation, DNA damage, and DNA repair inhibition
	LCA	Clostridium hiranonis, Clostridium hylemonae, Clostridium sordelli, Clostridium scindens	Apoptosis, cell proliferation, oxidative DNA damage, inflammatory reactions
	UDCA	*Parabacteroides distasonis*	Antiapoptotic, anti‐inflammatory, and antioxidant effect
SCFAs	Acetate	*Bacteroides. Hydrogenotrophica*, *M ethanobrevibacter smithii*, *R uminococcus gnavus*	–
	Butyrate	*Roseburia intestinalis*, *Butyrivibriocrossotus*, *Faecalibacterium cf. prausnitzii*	Immunity, inflammation, differentiation, apoptosis, phagocytosis, and efferocytosis
	Formate	*F. nucleatum*	AhR signaling, cancer stemness, inflammation
TMAO	TMAO	*Enterobacteriaceae*	Inflammation, altering protein folding, oxidative stress, and DNA damage
Tryptophan	Indole	*Escherichia coli, Bacteroides thetaiotaomicron, Bacteroides ovatus, C. limosum*	Suppress inflammation, fix abdominal wall structures, AHR ligends
	IAA	*Bacteroides ovatus, Bacteroides fragilis, Escherichia coli*	Suppress inflammation, fix abdominal wall structures, AHR ligends
Serine	S‐adenosylmethionine	*Saccharomyces cerevisiae, Pichia pastoris, Candida utilis*, and *Escherichia coli*	Upregulates cell adhesion gene expression and promotes cell migration and metastasis
Virulence factors	FadA	*Fusobacterium nucleatum*	mediates adhesion and invasion of epithelial and endothelial cells
	Fap2	*Fusobacterium nucleatum*	Immunity, binds to Gal‐GalNAc

BAs: bile acids; DCA: deoxycholic acid; LCA: lithocholic acid; UDCA: ursodeoxycholic acid; SCFAs: short‐chain fatty acids; TMAO: trimethylamine n‐oxide; IAA: indoleacetic acid.

### 3.1 Bile Acids

BA metabolism is associated with gut microbiota and exerts both beneficial and harmful effects on the host.^[^ ^89^ ^]^ Primary BAs, including cholic acid (CA) and chenodeoxycholic acid (CDCA), are produced using cholesterol in the liver, conjugated to glycine or taurine, and excreted into the duodenum to facilitate fat digestion. They are biotransformed and activate the gut microbiota by reshaping the gut microbiota composition.^[^ ^10b^ ^]^ On the one hand, BAs can alter membrane lipid composition, and increased BA concentrations can solubilize membranes and dissociate integral membrane proteins.^[^ ^90^ ^]^ On the other hand, BAs could also shape the composition of the gut microbiota by indirect effects through BA receptors, especially the farnesoid X receptor (FXR). In healthy participants, the FXR agonist obeticholic acid (OCA) inhibited the synthesis of endogenous BAs and significantly increased the abundances of *S. thermophilus*, *Lactobacillus casei*, and *Bifidobacterium breve*, suggesting that FXR activation alters the small intestinal microbiota in response to changes in endogenous BA concentrations.^[^ ^91^ ^]^ Subsequently, the BAs were reabsorbed in the distal ileum for enterohepatic circulation. BAs that enter the colon undergo complex biotransformation through interaction with gut microbiota, and are converted to secondary BAs,^[^ ^87^ ^]^ which are linked to tumor progression. Notably, accumulating evidence has demonstrated an association between BA metabolism and CRC.^[^ ^92^ ^]^ For example, CA and CDCA were found to regulate NF‐*κ*B and Janus kinase 2 (JAK2)/signal transducer and activator of transcription 3 (STAT3) signaling pathways to promote CRC.^[^ ^93^ ^]^ In addition, in N‐methyl‐N′nitro‐N‐nitrosoguanidine‐treated mice infusion of CA and CDCA caused a higher incidence of neoplasia in normal versus germ‐free mice, which highlights the key role of microbiota conversion of BAs in CRC progression.^[^ ^94^ ^]^

Bile salt hydrolases catalyze the hydrolysis of the conjugated primary BAs and have been purified from bacteria of the gut microbiota, including *B. fragilis*, *Bacteroides vulgatus*, *Clostridium perfringens*, *Listeria monocytogenes*, *Lactobacillus*, and *Bifidobacterium*.^[^ ^87^ ^]^ Dehydroxylation is also essential for secondary BA formation, and is catalyzed by intestinal bacterial enzymes.^[^ ^95^ ^]^

Both primary and secondary BAs are involved in the regulation of lipid activity and glucose metabolism, which is enabled by BA‐responsive receptors, including FXR, pregnane X receptor (PXR), constitutive androstane receptor, vitamin D receptor (VDR), and G protein‐coupled bile acid receptor 1 (TGR5).^[^ ^96^ ^]^ BA could activate these receptors and play an essential role not only in regulating lipid absorption and energy homeostasis, but also in initiating various pathologies, including CRC.^[^ ^97^ ^]^ The related BA‐responsive receptors and associated signaling pathways of the major BAs in CRC are summarized in **Table** **4**.

**Table 4:** Major bile acids in CRC and their related bile acid‐responsive receptors and associated signaling pathways

BAs	BA‐responsive receptors	Associated signaling pathways
CA	Not specified	NF‐*κ*B signaling, MAPK signaling
CDCA	TGR5	NF‐*κ*B signaling, JAK2/STAT3 signaling
DCA	FXR, PXR	NF‐*κ*B signaling, EGFR/MAPK signaling, Wnt/*β*‐catenin signaling, ERK signaling
LCA	VDR	NF‐*κ*B signaling, Wnt/*β*‐catenin signaling
UDCA	TGR5	YAP signaling, EGFR/ERK signaling

CA: cholic acid; CDCA: chenodeoxycholic acid; DCA: deoxycholic acid; LCA: lithocholic acid; UDCA: ursodeoxycholic acid; FXR: farnesoid X receptor; PXR: pregnane X receptor; VDR: vitamin D receptor; TGR5: G protein‐coupled bile acid receptor 1; NF‐*κ*B: nuclear factor‐kappa B; MAPK: mitogen‐activated protein kinase; JAK2: Janus kinase 2; STAT3: signal transducer and activator of transcription 3; EGFR: epidermal growth factor receptor; ERK: extracellular regulated kinase; YAP: yes‐associated protein.

#### 3.1.1 Deoxycholic Acid (DCA)

DCA, one of the main secondary BAs produced by *Clostridium*, is a tumor promoter in CRC.^[^ ^98^ ^]^ Clinical studies found a relationship between serum DCA levels and colonic mucosal proliferation,^[^ ^99^ ^]^ and more importantly, an increase in DCA levels in the sera and feces from patients with CRC.^[^ ^89^ ^]^ The role of DCA in CRC progression is attributed its regulatory effects on cellular signaling cascades and its membrane perturbing effects. DCA has detergent properties that can result in membrane perturbations, leading to prostaglandin and ROS production from arachidonic acid via the activation of COX‐2 and lipooxygenase. This results in inflammation, angiogenic formation, DNA damage, and DNA repair inhibition, which could be reversed through apoptotic promoting effect of PXR through DCA resistance.^[^ ^100^ ^]^ Additionally, the epidermal growth factor receptor and *β*‐catenin signaling pathway are activated in response to reduced expression of FXR,^[^ ^101^ ^]^ and *β*‐catenin stabilized COX‐2 mRNA to form a positive feedback loop.^[^ ^102^ ^]^ DCA was also found to specifically reduced adhesion of HET‐1A cells to vitronectin and reduced cell–surface expression of integrin‐*αν* via effects on endocytic recycling processes.^[^ ^103^ ^]^ Moreover, DCA regulates p53 through proteasome‐mediated degradation by stimulating the extracellular signal‐related kinase (ERK) signaling pathway, thereby enhancing tumor promotion in CRC.^[^ ^104^ ^]^

#### 3.1.2 Lithocholic Acid (LCA)

LCA, mainly produced by *Clostridium*, is a secondary BA that acts as an endogenous CRC promoter, similar to DCA.^[^ ^99^ ^]^ It produces ROS in the gastrointestinal tract, leading to destruction of the epithelial layer through decreased apoptosis, enhanced cell proliferation, oxidative DNA damage, inflammatory reactions, and NF‐*κ*B signaling pathway activation.^[^ ^89^ ^]^ Additionally, LCA modulates the muscarinic 3 receptor and Wnt/*β*‐catenin signaling in colonic epithelial cells, promoting cancer stemness that play a role in the development of CRC.^[^ ^105^ ^]^ Loss of the human leukocyte antigen was observed in colon adenocarcinoma cells after LCA treatment, and this may be an immune surveillance escape mechanism.^[^ ^106^ ^]^ Furthermore, LCA treatment induced increased the expression of matrix metalloproteinase (MMP) genes, including *MMP‐1*, *MMP‐2*, and *MMP‐7*, and stimulated urokinase plasminogen activator receptor, which induced tumor invasion and metastasis and promoted a malignant epithelial phenotype.^[^ ^107^ ^]^ IL‐8 expression was also enhanced when stimulated with LCA, thus, activating the ERK1/2/mitogen‐activated protein kinase (MAPK) signaling pathway, suppressing the activity of STAT3, and stimulating CRC angiogenesis.^[^ ^108^ ^]^ However, LCA was also determined to be CRC‐protective in a context‐dependent manner with the VDR. LCA was found to bind and activate VDR, inducing expression of CYP3A in vivo, which functions to detoxify BAs in the liver and intestine, thereby providing protection against CRC.^[^ ^109^ ^]^

#### 3.1.3 Ursodeoxycholic Acid (UDCA)

UDCA is a secondary BA and the 7*β*‐hydroxy isomer of CDCA produced by *Parabacteroides distasonis*, and used in clinical settings due to its therapeutic effects in cholesterol gallstone diseases and other digestive diseases, including biliary cirrhosis, sclerosing cholangitis, and recurrent pancreatitis.^[^ ^98^ ^]^ However, the effect of UDCA on CRC remains debatable.

UDCA exerts its therapeutic effects by upregulating the hydrophilicity of the biliary pool and downregulating the potency of harmful secondary BAs, including DCA and LCA which can lower inflammatory reactions on the colonic mucosa, through modulation of epidermal growth factor receptor/ERK signaling pathways.^[^ ^110^ ^]^ UDCA can also inhibit the activation of COX‐2 by DCA, thereby controlling inflammatory reactions.^[^ ^100a^ ^]^ In addition, UDCA was found to protect against malignant progression of CRC through TGR5–yes‐associated protein (YAP) axis.^[^ ^111^ ^]^

By contrast, other studies have claimed that UDCA can be converted into toxic LCA by microorganisms and lead to CRC development and progression. The authors argued that *Clostridium* can convert UDCA to LCA and impact the 7*α*‐dehydroxylation of CA and CDCA.^[^ ^112^ ^]^ Moreover, the protective effects of UDCA against colorectal adenomatous polyps were investigated, and UDCA had no significant overall effect on cancer risk reduction.^[^ ^112^ ^]^ Further studies on UDCA are warranted to determine its effect and underlying mechanisms in CRC.

### 3.2 SCFAs

SCFAs are metabolites found in the digestive system, which are produced from microbial fermentation of fiber by anaerobic gut microbiota, including Firmicutes (*Clostridium* and *Lactobacillus*), Bacteroides (*Bifidobacterium*), Actinobacteria (*Propionibacterium*), Proteobacteria, and Verrucomicrobia.^[^ ^113^ ^]^ SCFAs are easily absorbed and can supply energy to colon cells.^[^ ^114^ ^]^ Butyrate, propionate, and acetate are the three main SCFAs in the intestines; butyrate is primarily produced by Firmicutes,^[^ ^115^ ^]^ propionate by Firmicutes, Bacteroides, *Acetobacterium*, and *Clostridium aceticum*, and acetate by *Propionibacterium* species.^[^ ^116^ ^]^ Importantly, SCFAs are a key component of glycolysis that play important roles in host and cell homeostasis,^[^ ^117^ ^]^ act as protective molecules in CRC development, and change as the gut microbiota changes during tumor progression;^[^ ^11b^ ^]^ therefore, SCFAs are closely associated with CRC development.

#### 3.2.1 Butyrate

Butyrate is produced by anaerobic bacteria (such as *Roseburia intestinalis*, *Butyrivibriocrossotus*, *Faecalibacterium cf. prausnitzii*) in the colon,^[^ ^118^ ^]^ provides energy for colorectal epithelial cells,^[^ ^119^ ^]^ and inhibits inflammation and tumor formation.^[^ ^120^ ^]^ Expression of a butyrate transporter protein, which could maximize the transport and metabolism of butyrate induced by rapid beta‐oxidation to generate cellular fuel,^[^ ^121^ ^]^ was reduced in tumors of patients with CRC.^[^ ^122^ ^]^ Notably, epithelial barrier dysfunction is a major contributing factor to the development and progression of CRC because it triggers inflammation by activating macrophages that produce inflammatory cytokines.^[^ ^123^ ^]^ Butyrate can help to maintain epithelial tight junctions through activation of adenosine monophosphate‐activated protein kinase^[^ ^124^ ^]^ and protect the function of the colon epithelial barrier through induction of mucin 2 secretion,^[^ ^125^ ^]^ which may occur in a GPR43‐dependent manner.^[^ ^126^ ^]^ Moreover, butyrate can affect the internal nervous system and regulate neuronal excitability; thus, colon motility and extracorporeal contraction reactions are increased, which prevents CRC development.^[^ ^127^ ^]^

Butyrate also accumulates and functions as a histone deacetylase (HDAC) inhibitor to accelerate histone acetylation and participate in the apoptosis and proliferation of CRC cells.^[^ ^128^ ^]^ Additionally, butyrate impedes the angiogenesis, metastasis, and survival of CRC cells through the neuropilin‐1/vascular endothelial growth factor pathway by inhibiting *Sp1* transactivation;^[^ ^129^ ^]^ it can reduce cell proliferation, clone formation, and migration by promoting secretion of endocan through the ERK2/MAPK pathway;^[^ ^130^ ^]^ butyrate inducing apoptosis in CRC cells by inhibiting the Wnt/*β*‐catenin signaling^[^ ^131^ ^]^ and facilitates anticancer therapy efficacy by modulating cytotoxic CD8^+^ T cell immunity.^[^ ^132^ ^]^ In summary, butyrate acts as a vital gut microbiome metabolite and protects against the progression of CRC.

#### 3.2.2 Other SCFAs

Acetate and propionate are SCFAs fermented from soluble fibers, which have protective effects against CRC. As the final product of fermentation, acetate can be condensed to butyrate in the human colon by bacteria, such as *Roseburia spp*., *F. prausnitzii*, and *Coprococcus sp*. through the action of butyryl‐CoA:acetate CoA‐transferase,^[^ ^133^ ^]^ thereby contributing to the syntrophy of the gut microbiome and inhibiting CRC progression. Propionate is an HDAC inhibitor which inhibits malignant transformation and induces the apoptosis of precancerous colonic cells, despite its poor effectiveness (in comparison to butyrate) and reduced abundance in CRC.^[^ ^134^ ^]^ Finally, formate secreted by *F. nucleatum*, can drives CRC tumor invasion by triggering Aryl hydrocarbon receptor signaling, while increasing cancer stemness.^[^ ^135^ ^]^ Formate can be considered a gut‐derived oncometabolite relevant for CRC progression.

### 3.3 Trimethylamine n‐oxide (TMAO)

TMAO is a small organic compound, mainly produced by *Enterobacteriaceae*, whose concentration in blood increases after ingestion of dietary l‐carnitine and phosphatidylcholine. TMAO is a marker of cardiovascular risk associated with CRC. A prospective cohort study of 835 CRC cases and 835 matched controls found a positive correlation between plasma TMAO levels and CRC risk for the first time. The study showed that subjects with high plasma TMAO levels had a 3.4‐fold higher risk of CRC than subjects with low plasma TMAO levels.^[^ ^136^ ^]^ In 2017, a study found that serum TMAO levels in patients with CRC was significantly higher than in healthy control subjects. In addition, the study reported that patients with higher serum TMAO levels have lower survival rates than those with lower serum TMAO levels, thus, serum TMAO is a possible prognostic marker for CRC.^[^ ^137^ ^]^ Although these studies suggest a direct or indirect role for TMAO in CRC, others have shown no correlation between serum TMAO levels and CRC risk. A study conducted on 677 CRC patients and 677 healthy controls reported no significant correlation between serum TMAO levels and the incidence of CRC.^[^ ^138^ ^]^ Since TMAO levels vary by ethnicity, diet, and region, larger clinical trials are needed to validate the TMAO–CRC correlation, and whether elevated TMAO levels are a cause or a consequence of cancer. Recent studies have postulated a possible association between TMAO levels and CRC, but there is no valid mechanistic approach to its role in carcinogenesis. As studied previously, TMAO may interact in several processes that could impact CRC, such as inflammation, protein folding, oxidative stress, and DNA damage.^[^ ^139^ ^]^ Excess TMAO leads to sustained and uncontrolled ROS production, which causes inflammation. Inflammation can damage DNA, cause mutations or induce genomic instability, and ultimately leading to the development of CRC.^[^ ^139^ ^]^

### 3.4 Amino Acids

When compared to healthy controls, 17 metabolites were significantly altered in CRC patients.^[^ ^140^ ^]^ Among them, 10 metabolites are amino acids, specifically l‐alanine, glycine, l‐proline, l‐aspartate, l‐valine, l‐leucine, l‐serine, l‐phenylalanine, l‐*α*‐aminobutyric acid, and norvaline. Furthermore, differences in the pathways associated with branched‐chain amino acid (BCAA) metabolism in the stages leading to CRC were also observed. Compared to healthy control, the first 4 enriched pathways in CRC are 1) amyl‐tRNA biosynthesis, 2) valine, leucine, and isoleucine biosynthesis, 3) phenylalanine metabolism, and 4) phenylalanine, tyrosine, and tryptophan biosynthesis, suggesting that amino acids metabolic pathways other than individual amino acids in the colorectum are altered in the early the stages leading to CRC.^[^ ^140^ ^]^

The metabolism of amino acids provides the host with biologically active metabolites through gut microbiota. The amino acid tryptophan is catabolized to a series of indole derivatives (e.g., indole‐3‐acetic acid, indole‐3‐sulfate, indole‐3‐propionic acid, and indole‐3‐aldehyde), which are ligand receptors for aromatic hydrocarbons, such as the Aryl hydrocarbon receptor. Activation of innate lymphocytes and aromatic hydrocarbons in gut‐resident T cells increases IL‐22 production, which helps to prevent colonic inflammation (colitis) and inflammatory bowel disease, which can lead to precancerous lesions.^[^ ^141^ ^]^ In addition, in vivo, tryptophan metabolites not only suppress inflammation, but also fix abdominal wall structures and in association with beneficial bacteria in the gut, may slow the progression of CRC.^[^ ^142^ ^]^ Tryptophan metabolism plays an important role in inhibiting the growth of CRC, although the underlying mechanism remains nebulous.

Serine metabolism plays a crucial role in the proliferation and survival of tumor cells. Phosphoglycerate dehydrogenase (PHGDH), a key enzyme in serine biosynthesis, can undergo monoubiquitination and increase activity in CRC cells, thereby increasing levels of serine, glycine, and S‐adenosylmethionine (SAM), which in turn upregulates cell adhesion gene expression and promotes cell migration and CRC metastasis.^[^ ^143^ ^]^

### 3.5 Other Metabolites

In addition to the above, metabolites originating from dietary phytochemicals also play important roles in CRC. Most of the dietary phytochemicals are transformed into other metabolites by the gut microbiota in the colon through hydrogenation, dehydroxylation, and demethylation.^[^ ^144^ ^]^ These metabolites have altered bioactivity, and some phenolic metabolites, such as the stilbene resveratrol and dehydroxylated phenolic acids, have anti‐inflammatory properties, thus, reducing the secretion of inflammatory cytokines, such as TNF‐*α*, COX‐2, and NF‐*κ*B.^[^ ^145^ ^]^ Moreover, a few phenolic compounds produced by the gut microbiota have antimicrobial effects and alter the composition of gut microbiota by suppressing pathogens such as *C. perfringens*, and promoting probiotics such as *Lactobacilli spp*.,^[^ ^146^ ^]^ thereby producing knock‐on effects on gut microbiota metabolism.^[^ ^16a^ ^]^ Besides, some virulence factors, such as *Fusobacterium* adhesin A (FadA) and fibroblast activation protein 2 (Fap2), are induced by gut microbiota and contribute to carcinogenesis. FadA, secreted by *F. nucleatum*, is a highly conserved virulence factor in *C. perfringens* that mediates adhesion and invasion of epithelial and endothelial cells.^[^ ^47^ ^]^ The binding of FadA to E‐cadherin activates the *β*‐catenin signaling pathway, which in turn increases NF‐*κ*B, Wnt7b, and the cyclin D1 and Myc oncogenes.^[^ ^42^ ^]^ Fap2 is a galactose‐sensitive hemagglutinin and adhesin that may play a role in the virulence of *C. perfringens*.^[^ ^147^ ^]^ The binding of Fap2 to the T‐cell immune receptor with immunoglobulin G inhibits the activity of natural killer cells against tumor cells, leading to the development of CRC.^[^ ^148^ ^]^ Abed et al. showed that fusobacterial Fap2 could act as a lectin, binding to d‐galactose‐*β*‐(1–3)‐N‐acetyl‐d‐galactosamine, which is overexpressed in CRC.^[^ ^149^ ^]^ Investigations on fecal metabolites associated with mouse models of colon tumorigenesis with varying mutational loads revealed that *Lactobacillus reuteri* and its metabolite, reuterin, are downregulated in mouse and human CRC. Reuterin may alter the redox balance and reduce proliferation and survival of colon cancer cells or induce selective protein oxidation and inhibit ribosomal biogenesis and protein translation to protect against CRC.^[^ ^150^ ^]^

Several gut microbiota‐derived metabolites contribute to CRC development and progression. Thus, a better understanding of the gut microbiota pathways involved in the biosynthesis of CRC‐related metabolites would greatly aid in intestinal health management, especially CRC prevention. It is important to identify the causal role between gut microbiota‐derived metabolites and CRC.

### 3.6 Role of Diet on the Gut Microbiota–Host Interactions

As discussed above, the gut microbiota may influence the pathophysiology of host disease through several pathways, including the secretion of metabolites.^[^ ^33^ ^]^ In addition, a direct correlation between diet and the type and amount of gut bacteria has been reported.^[^ ^151^ ^]^ The impact of diet on gut microbiota–host interactions is increasingly recognized and explored (**Table** **5**). The Western diet is characterized by high levels of protein, sugar, saturated fat, refined grains, alcohol, salt, high‐fructose corn syrup, and low levels of fiber.^[^ ^152^ ^]^ It has been found to induce microbial dysbiosis, increasing the abundance of *Fusobacterium* spp., *E. coli*, and *Enterobacter* spp. and decreasing the abundance of *Faecalibacterium prausnitzii*, *Roseburia hominis*, and *Bifidobacterium* spp., thus leading to inflammatory responses in the host,^[^ ^153^ ^]^ which increases the risk of CRC. A plant‐based diet has been found to increase the abundance of *Bifidobacterium* spp. and *Lactobaccilus* spp. and decrease the abundance of *B. fragilis* and *C. perfringens*, thereby reducing inflammatory responses.^[^ ^154^ ^]^ Foxtail millet consumption has been shown to increase the abundance of *Bifidobacterium* spp. and *Bacteroidales*_S‐24‐7, thereby attenuating colonic inflammation and reducing colitis‐associated CRC in mouse models.^[^ ^155^ ^]^ Research into the effect of diet on CRC‐associated gut microbiota–host interactions is still lacking. Given that diet is the most important factor influencing the dynamics of the gut microbiota, comprehensive studies of gut microbiota–host interactions shaped by diet have the potential to provide insights into the mechanisms underlying CRC.

**Table 5:** Diet‐correlated gut microbiota–host interactions

Diet	Gut microbiota		Host interaction
	Increase	Decrease	
Western diet	*Fusobacterium* spp. *Escherichia coli* *Enterobacter* spp.	*Faecalibacterium prausnitzii* *Roseburia hominis* *Bifidobacterium* spp.	Proinflammatory
Plant‐based diet	*Bifidobacterium* spp. *Lactobaccilus* spp.	*Bacteroides fragilis* *Clostridium perfringens*	Anti‐inflammatory
Foxtail millet consumption	*Bifidobacterium* spp. *Bacteroidales*_S‐24‐7	∖	Anti‐inflammatory

## 4 Potential Roles of Gut Microbiota and Its Metabolites in CRC Diagnosis and Treatment

**Figure 4:**
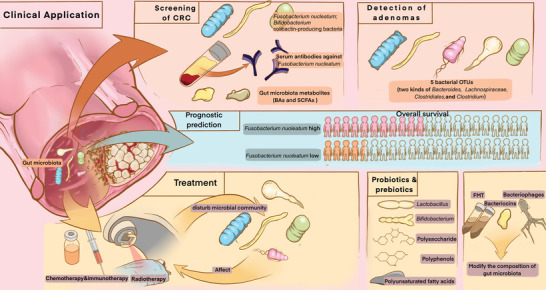
The role of gut microbiota in CRC diagnosis and treatment. Detection of fecal bacteria including *F. nucleatum*, serum antibodies against *F. nucleatum* and gut microbiota metabolites including BAs and SCFAs could play roles in CRC screening. Fecal bacteria including two species of *Bacteroides*, *Lachnospiraceae*, *Clostridiales*, and *Clostridium* could be used to detect adenomas. *F. nucleatum* can be used in prognostic predictions for CRC patients. Treatment methods including chemotherapy, immunotherapy, and radiotherapy could disturb microbial community, while the composition of the gut microbiota could, in turn, affect the treatments. Methods including supplementation with probiotics, prebiotics and their metabolites, and fecal microbiota transplantation (FMT) could modify gut microbiota composition in order to treat CRC.

Owing to the dysbiosis of gut microbiota, several species are involved in CRC diagnosis and treatment, which could be used as therapeutic targets. Herein, we summarized the applications of gut microbiota in screening, prognosis, and treatment of CRC (**Figure** **4**).

### 4.1 Role of Gut Microbiota in CRC Diagnosis

Due to the emerging clinical demand of CRC screening and treatment, which remarkably improves CRC prognosis,^[^ ^156^ ^]^ research and development on accurate and noninvasive diagnostic methods are of necessity. In addition to traditional (endoscopy) and nascent (fecal immunochemical test (FIT) and circulating tumor DNA) diagnostic methods,^[^ ^157^ ^]^ fecal microbial markers are a promising and desirable diagnostic tool as they seem accurate, economical, and noninvasive.^[^ ^6^ ^]^

#### 4.1.1 CRC Screening

Early detection of CRC through screening has been widely accepted as a main path in CRC prevention and treatment, and it significantly improves the overall survival rate of patients.^[^ ^158^ ^]^ Fecal samples are most ideal for screening because of the noninvasive collection method. FIT has been applied in clinical settings in recent years, but its sensitivity for CRC is limited.^[^ ^159^ ^]^ FIT using fecal DNA has >90% sensitivity in CRC detection, but <50% sensitivity in detecting advanced colorectal adenomas.^[^ ^160^ ^]^

Fecal microbial markers are being considered to screen patients for CRC with enhanced sensitivity and accuracy. Based on microbiota‐based screening, several bacterial species with alterations in abundance were selected and compared to FIT and a fecal occult blood test.^[^ ^161^ ^]^ Among the selected bacteria, addition of *F. nucleatum* screening enhanced the accuracy and sensitivity of FIT,^[^ ^161^, ^162^ ^]^ which reached a sensitivity and specificity of 84.6% and 92.3%, respectively, when combined with *Bifidobacterium* ^[^ ^163^ ^]^ and a sensitivity and specificity of 84.6% and 63.1%, respectively, when combined with colibactin‐producing bacteria.^[^ ^164^ ^]^ In addition, serum antibodies against *F. nucleatum* were also useful in CRC detection.^[^ ^165^ ^]^ Importantly, detection of adenomas is also a key factor in CRC prevention. Combination of the abundance of five bacterial species and other clinical parameters reached an AUC of 0.90, indicating the potential value of fecal microbial markers in colorectal adenoma detection.^[^ ^166^ ^]^ Interestingly, an increased abundance of *F. nucleatum* was also detected in the fecal samples of patients with adenomas, and this can be evaluated in further studies.^[^ ^161b^ ^]^

Additionally, gut microbiota metabolites have the potential in screening for CRC. Divergence of BAs and SCFAs were determined in fecal extracts.^[^ ^167^ ^]^ Higher levels of acetate, valeric acid, isobutyric acid, and isovaleric acid, and lower levels of butyrate, linoleic acid, glycerol, oleic acid, and trans‐oleic acid were detected in patients with CRC than in healthy individuals.^[^ ^167^ ^]^ However, further large‐scale clinical studies should be conducted to investigate the feasibility of CRC screening using fecal microbial markers, gut microbiota, and its metabolites.

#### 4.1.2 Prediction of CRC Prognosis

Fecal microbial markers are correlated to the clinical outcomes of CRC and are useful in predicting CRC prognosis.^[^ ^25^ ^]^ *F. nucleatum* is a key factor in prognostication; its abundance in CRC tumors is negatively correlated with patients’ survival.^[^ ^168^ ^]^ Importantly, further research on the value of bacterial markers in CRC prognostication must be conducted, with a wider range of microorganisms and limited interference factors.

### 4.2 Role of Gut Microbiota in CRC Treatment

Chemotherapy and immunotherapy have dramatically improved the survival of patients, but the side effects and sequelae have become increasingly significant. These therapies disturb the gut microbiota leading to dysbiosis which can be reversed after treatment.^[^ ^169^ ^]^ More importantly, the gut microbiota can enhance treatment efficacy and reduce adverse effects.^[^ ^170^ ^]^

#### 4.2.1 Chemotherapy

Chemotherapy disturbs the microbial community, leading to a reduction in microbial diversity and changes in the abundance of several bacteria.^[^ ^171^ ^]^ For example, treatment with 5‐fluorouracil (5‐FU) can result in the enrichment of Proteobacteria, and decrease of Firmicutes and Actinobacteria, resulting in a reduction of inflammatory reactions through interaction with the NF‐*κ*B signaling pathway and production of SCFAs.^[^ ^172^ ^]^ Conversely, the gut microbiota can also affect chemotherapy within a mechanistic framework: translocation, immunomodulation, metabolism, enzymatic degradation, and reduced diversity and ecological variation (TIMER).^[^ ^169^ ^]^ The curative effects of many widely applied agents, including 5‐FU,^[^ ^172^ ^]^ gemcitabine,^[^ ^173^ ^]^ oxaliplatin,^[^ ^174^ ^]^ and cyclophosphamide,^[^ ^175^ ^]^ are regulated by gut microbiota using the TIMER mechanism. In vivo studies have proven that a lack of gut microorganisms can diminish the therapeutic effects of drugs on tumor‐infiltrating myeloid‐derived cells and lead to ROS production and cytotoxicity.^[^ ^174^ ^]^ Notably, *F. nucleatum* has been shown to enhance the resistance of colonized tumors to the therapeutic effects of 5‐FU and oxaliplatin through the activation of cell autophagy.^[^ ^40^ ^]^ Therefore, chemotherapy can be augmented by therapeutic agents targeting *F. nucleatum* to improve treatment outcomes, and detection of this bacterium may predict the potency of chemotherapy, thus, it should be considered in the future.

#### 4.2.2 Immunotherapy

Inhibitory signals of T cell activation can be reversed by immune checkpoint inhibitors (ICIs) to enable tumor‐reactive T cells to induce significant antitumor responses.^[^ ^176^ ^]^ This type of immunotherapy has not been widely applied in clinical settings but has attracted great attention as a promising therapeutic pathway in CRC treatment,^[^ ^177^ ^]^ especially in patients with CRC who have high levels of microsatellite instability and/or DNA mismatch repair‐deficient metastasis.^[^ ^178^ ^]^ Interestingly, immunotherapy alters the composition of the gut microbiota,^[^ ^179^ ^]^ which has a role in the immune responses generated by immunotherapy.^[^ ^180^ ^]^ The gut microbiota regulates a few pivotal ICIs, including the programmed cell death 1 (PD‐1)/PD‐1 ligand 1^[^ ^181^ ^]^ and cytotoxic T lymphocyte‐associated antigen 4 axes.^[^ ^182^ ^]^ Several bacteria, including *B. fragilis*,^[^ ^182a^ ^]^ *Akkermansia muciniphila*,^[^ ^181a^ ^]^ *Alistipes shahii*,^[^ ^174^ ^]^ *Bifidobacterium spp*.,^[^ ^181b,d^ ^]^ *Faecalibacterium spp*.,^[^ ^183^ ^]^ and *Eubacterium limosum*,^[^ ^184^ ^]^ are advantageous to immunotherapy. Bacterial supplements of *Lactobacillus*, *Ruminococcus*, and *Alistipes* can enhance the therapeutic response to ICIs,^[^ ^174^ ^]^ and bacteria, such as *Akkermansia muciniphila*, accumulate in immunotherapy responders.^[^ ^185^ ^]^ However, some of the side effects of immunotherapy are correlated with the gut microbiota,^[^ ^182a^ ^]^ and resistance to immunotherapy is associated with several bacteria, including *Bacteroidetes*.^[^ ^186^ ^]^ Therefore, additional treatment targeting these bacteria might be conducive to the therapeutic efficacy of immunotherapy toward cancers, including CRC.^[^ ^174^, ^187^ ^]^

#### 4.2.3 Probiotics and Prebiotics

With the increasing awareness of the importance of gut microbiota in tumorigenesis and cancer treatment outcomes, restoring intestinal microbiota homeostasis has become a potential CRC prevention and treatment measure. Probiotics and prebiotics are emerging biotherapeutics, which modify the gut microbiota, thus, benefiting the host.^[^ ^188^ ^]^

Probiotics are microorganisms which benefit the human body if taken in moderation.^[^ ^188^ ^]^ *Lactobacillus* and *Bifidobacterium* are the most studied probiotics in CRC treatment.^[^ ^189^ ^]^ As the concept of probiotics evolved over the past few decades, it is now understood that the effects of probiotics are not only microbiota‐mediated, but also host‐mediated through the induction of physiological and metabolic changes. Probiotic administration corrects microbial dysbiosis and maintains the intestinal microbial balance by occupying host tissues, preventing colonization by pathogenic bacteria, exerting immunomodulatory effects, and enhancing gut barrier function.^[^ ^190^ ^]^ Evidence has indicated that probiotics could diminish tumor incidence and act as a preventive treatment for CRC. For instance, *Lactobacillus plantarum* decreased tumor development by increasing the innate immune response through a set of reactions, including dendritic cell maturation which led to the polarization of Th1 responses, migration of CD8^+^ and natural killer cells, reduction of tumor growth, and prolongation of survival of mice with CRC.^[^ ^191^ ^]^ Furthermore, clinical trials have indicated that a lower incidence of moderate and higher atypia‐graded tumors were found in patients receiving a preparation of *Lactobacillus casei* than in patients not receiving this probiotic.^[^ ^192^ ^]^

Prebiotics are substrates which are selectively utilized by host microorganisms that confer health benefits.^[^ ^188^ ^]^ At present, prebiotics are predominantly carbohydrate‐based, but other substances, such as polyphenols and polyunsaturated fatty acids, may also play a prebiotic role.^[^ ^188^ ^]^ Prebiotics exert their beneficial health effects in the intestines mainly by stimulating the growth and selective fermentation of probiotics, interacting with pathogens and preventing colonization, playing an anti‐inflammatory role, exerting selective cytotoxicity on tumor cells, inhibiting the apoptosis of normal epithelial cells, and enhancing IgA secretion that protects the intestinal epithelium.^[^ ^190^ ^]^ Butyrate treatment promotes epithelial cell proliferation, polyp formation, and ultimately leads to tumor progression in Apc^min/+^; *Msh2* ^−/−^ mice.^[^ ^193^ ^]^ Though the strength of evidence for prebiotic interventions lags behind that for probiotics, prebiotics may improve the abundance of four symbiotic microbes, which can be opportunistic pathogens in patients with CRC.^[^ ^194^ ^]^

Although the results of animal experiments are highly encouraging, the quantity and quality of clinal trials related to this field remains limited. It has been extremely challenging to conduct randomized clinical trials with large sample sizes, proper randomization, analysis, and validation to obtain sufficient evidence to support the benefit of prebiotic treatment in CRC.

#### 4.2.4 Other Microbiota‐Related Therapeutic Approaches

Other microbiota‐related therapeutic approaches which may be effective against CRC are also being considered. These include antibiotics and fecal microbiota transplantation (FMT). Antibiotic treatment may kill intestinal microbiota and reverse harmful dysbiosis primarily through the elimination of carcinogenic B. fragilis, mucin degradation, inflammation, and DNA methylation.^[^ ^195^ ^]^ Metronidazole treatment reduced cancer cell proliferation and shrunk tumor volume in mice bearing a colon cancer xenograft.^[^ ^78^ ^]^ Another study also reported that an antibiotic cocktail composed of ampicillin, neomycin, metronidazole, and vancomycin depleted the gut microbiota and attenuated colon tumor formation in mice fed a high‐fat diet.^[^ ^196^ ^]^ However, the role of antibiotic treatment in cancer management is controversial as a register‐based study showed a robust association between antibiotic treatment and an increased risk of proximal CRC.^[^ ^197^ ^]^

FMT from healthy donors to individuals with cancer restores microbial homeostasis, which may be helpful for improving various gastrointestinal diseases, including irritable bowel syndrome, *Clostridium difficile* infection, and CRC.^[^ ^198^ ^]^ FMT also increases microbial diversity without disruption of the microbial gut ecology in contrast to antibiotic treatment, and its long‐term implantation allows it to be designed as a single‐dose regimen, thus, adding therapeutic benefits to probiotics and prebiotics, whose effects are short‐term.^[^ ^199^ ^]^ A recent study reported that FMT from wild mice to laboratory mice improved host fitness and resistance against AOM/DSS‐induced colorectal tumorigenesis.^[^ ^200^ ^]^ The practical application of FMT in patients with CRC awaits trial results on its effect (in combination with cancer immunotherapy or chemotherapy), which are in progress.^[^ ^201^ ^]^ Other novel approaches could include bacteriocins^[^ ^202^ ^]^ or bacteriophages^[^ ^203^ ^]^ to modify the gut microbiota. More studies are imperative to evaluate the clinical application potential of these engineered products in CRC.

## 5 Conclusions and Outlook

Differences in the composition and abundance of the gut microbiota in patients with CRC patients and interaction between the gut microbiota and CRC have been reported. The gut microbiota and its metabolites interact closely with host epithelial cells and have a crucial role in CRC. As research elucidates the role played by intestinal flora during carcinogenesis, it offers unprecedented opportunities to explore applications in CRC diagnosis, treatment, and prognosis, although many obstacles remain.

Some questions are still to be answered by future research. Evidence such as the increased abundance of *F. nucleatum*, *E. coli*, or *B. fragilis* in CRC provides an opportunity for these organisms to be targeted as biomarkers in conjunction with conventional diagnostics. However, due to the diversity of sequencing methods, it is difficult to reach a consensus on the composition of the gut microbiota. Therapeutic approaches targeting the microbiome are gaining attention in clinical applications, yet their implementation remains challenging due to the numerous effects of the gut microbiota on host biology, many of which are currently unknown. More importantly, it should be of great concern that lifestyle, drugs, genetics, and other factors could have a significant impact on the remodeling of the gut microbiota. However, these influencing factors have been ignored and not corrected in many cancer studies, thus confounding the real facts and leading to potentially mistaken conclusions. Care should be taken to control for confounding factors in order to find the key bacteria and mechanisms that really influence the pathogenesis of cancers, including CRC, when analyzing the complex and diverse sequencing data of large sample size. For the metabolites, there is still a lack of evidence on the source of the metabolites detected in feces or serum, and there is still a debate on whether they are secreted by the gut microbiota or the host, or whether they are ingested from the diet. Clarification of metabolite sources will facilitate research into subsequent treatment modalities for CRC.

Exploring the significance of clinical transformation is an important direction for future microbial research. There could be three major potential applications of gut microbiota in CRC, including noninvasive diagnostic tools, restoration of microbial dysbiosis or probiotic supplementation for CRC prevention, or modulation of gut microbes to enhance therapeutic responses. Bacteria‐based therapy could be achieved by selective elimination of oncogenic microbes, fecal transplantation of antitumor species, and altering the composition of the microbiota through oral probiotic supplementation or targeted phage therapy. However, large‐scale clinical trials based on rigorous animal studies are needed to further establish their efficacy and safety in the clinic. Thus, such applications still remain a long way from routine use.

To proclaim the expected clinical potential of the gut microbiota in CRC, researchers must decipher whether specific strains or the collective capabilities of some microbiota, influence the development of disease. Further studies are needed to help fully determine the pathogenic role of the gut microbiota in carcinogenesis and its response to treatment, and to address the challenges that impede the achievement of personalized medicine, by focusing on specific individuals including early‐onset patients. With the vigorous development in this rapidly developing field, gut microbiota will become a crucial part of future diagnosis, cancer prevention, and treatment measures.

## Conflict of Interest

The authors declare no conflict of interest.

## Author Contributions

R.Q., Y.Z., and Y.M. contributed equally to the work. All authors have contributed to the article and approved its publication. R.Q., Y.Z., Y.M., X.Z., and L.S.: designing, writing, and figure preparation. L.S., C.J., Z.Z., and W.F.: designing, funding acquisition, reviewing, and editing.
